# The Assessment of the Spectrum of Preventive Measures Taken by Healthcare Providers During the COVID-19 Pandemic in India: A Survey-Based Study

**DOI:** 10.7759/cureus.41073

**Published:** 2023-06-28

**Authors:** Niraj Srivastava, Santosh K Rathia, Chandan Dey, Arvind Shukla, Pugazhenthan T, Sunita Singh, Varun Anand

**Affiliations:** 1 General Surgery, All India Institute of Medical Sciences, Raebareli, IND; 2 Trauma and Emergency (Pediatric Emergency Medicine), All India Institute of Medical Sciences, Raipur, IND; 3 Trauma and Emergency Medicine, All India Institute of Medical Sciences, Raipur, IND; 4 Community and Family Medicine/Biostatistics, All India Institute of Medical Sciences, Raipur, IND; 5 Pharmacology and Therapeutics, All India Institute of Medical Sciences, Raipur, IND; 6 Pediatric Surgery, All India Institute of Medical Sciences, Raebareli, IND

**Keywords:** unani, homeopathy, ayurvedic, pandemic, allopathy, preventive measures, prophylaxis, hcps, hcws, covid-19

## Abstract

Introduction: The first wave of the coronavirus disease 2019 (COVID-19) pandemic created havoc and confusion in choosing appropriate treatment, as well as prophylaxis, due to its rapid surge, disease novelty, and lack of evidence-based literature. It was even more concerning among the healthcare workers (HCWs), who had to take care of patients, themselves, and their own families.

Objective: This online survey-based study targeted finding the various options for COVID-19 precautionary or prophylactic measures opted for by HCWs.

Methodology: This was an observational study based on a predesigned questionnaire, which was floated online for three months after institutional ethical approval, just after the first wave of COVID-19 in 2021, targeting HCWs of different cadres (doctors, nurses, paramedics/laboratory technicians, etc.), ages, and sexes and HCWs hailing from across the country. Questions were focused on HCW’s adopted measures, the order of preference and its reasons, and concerns related to safety and efficacy. Data was collected through Google Forms (Google, Inc., Mountain View, CA) into an Excel spreadsheet (Microsoft^®^ Corp., Redmond, WA) and analyzed by the latest Statistical Package for Social Sciences (SPSS) software (IBM SPSS Statistics, Armonk, NY) using appropriate statistics.

Results: The routine practice of standard precautionary measures (face mask, hand hygiene, and social distancing) and wearing a personal protective equipment (PPE) kit during the care of COVID-19-positive or COVID-19-suspected patients was adopted by the majority of HCWs, i.e., 306/312 (>98%) irrespective of cadre (p = 0.001). After the “routine measures,” the most adopted measure by participant HCWs irrespective of profession, age, and gender was the consumption of allopathic drugs (n = 188; 60.26%). Anti-COVID-19 measures in the category of drugs used by healthcare providers (HCPs) were prophylactic allopathic drugs (60.26%), homeopathic drugs (11.86%), and other Ayurveda, Yoga, Naturopathy, Unani, Siddha, and Homeopathy (AYUSH)/traditional medical system drugs (11.86%). Vitamin C was the most consumed among all of the drugs for COVID-19 prophylaxis purposes. Non-drug measures espoused by the HCPs were physical exercises (46.47%), increased sleep duration (35.89%), change in dietary habits (42.62%), and spiritual measures (19.23%).

Conclusion: The fear of COVID-19 imposed on the HCPs the obligation to use all the available preventive measures in spite of the lack of evidence on actual benefits. After the routine infection preventive measures, the most adopted measure by participant HCWs irrespective of profession, age, and gender was the consumption of prophylactic allopathic drugs (>60%), and the most non-drug preventive measures were the initiation of physical exercises and change in dietary habits. Adapting some form of physical exercise was more noted with males than females (p = 0.001), and it significantly increased with HCPs of higher age of >25 and >40 years than younger HCPs (58.6% versus 29.3%; p = 0.016). Females preferred more dietary and nutritional modifications.

## Introduction

The first wave of coronavirus disease 2019 (COVID-19) infection was one of the deadliest infective pandemics that created trepidation not only in the lay population but also among healthcare providers (HCPs). During the first wave of COVID-19, anecdotal reports were available online through published literature, and some nonproven reports or statements were disseminated via social media, creating a situation of confusion among people. Because of its novelty and severity, the world started using all knowledge systems available globally, often without applying logic [1]. HCPs too adopted all possible ways to protect themselves from COVID-19, including, but not limited to, homeopathic, Ayurvedic, Unani, allopathic, and even traditional or ancient wisdom.

The side effects of some drugs hypothesized to prevent COVID-19 infection were also opted for as therapeutic options after considering their benefit-to-risk ratio. This resulted in the adoption of many non-pharmacological measures by HCPs to enhance their immunity, physical strength, and sense of protection from COVID-19 infection. Along with multiple governing and scientific body guidelines, a comprehensive preventive evaluation method has also been formulated and tested [2]. In the current study, the spectrum of preventive measures taken in various forms and interconnected justifications considered by participant HCPs to protect them from acquiring COVID-19 infection were assessed.

## Materials and methods

The study was carried out by the Department of Trauma and Emergency Medicine of the All India Institute of Medical Sciences (AIIMS), Raipur, over three months after getting clearance from the institutional research cell and institutional ethics committee (IEC number: AIIMSRPR/RC(P)/2020/208). The participants in the study were HCPs (nursing officers, doctors, hospital attendants or ward boys, laboratory technicians, dressers, and pharmacists) who consented to participate in the study and were working at different hospitals across the country. Thus, HCPs were the inclusion criteria. A questionnaire and a scoring system were designed by a team of clinical experts working at the Department of Trauma and Emergency Medicine at AIIMS, Raipur. To validate the questionnaire, the validating experts needed to verify all the points in the scoring system. All the criteria of the scoring system were agreed upon by all the experts independently. The “Hindi” language portion of the questionnaire was validated by other experts who were clinical experts and also had intermediate school certification in “Hindi” with “Hindi” as their mother tongue language. The Delphi method via electronic media was used for communication among the independent experts to share the suggested modifications in the questionnaire. The validated questionnaire was sent to all the study participants via an electronic link (web link), and the first page was enclosed as consent. A set of 10 questions, with additional sub-questions, were incorporated into the questionnaire. All the questions were designed to be open-ended, allowing the participants to express their opinions freely. When the available response options did not cover all possible choices, the participants were offered an “others” option to accurately and impartially convey their preferred alternatives. Incompletely filled electronic forms were rejected as part of the exclusion criteria, and the collected data was transformed into a statistical configuration for a definitive result and its valid representation.

## Results

A total of 312 HCPs participated in this survey-based observational study, and out of them, the number of females and males was 114 (36.5%) and 198 (63.5%), respectively. The participants were categorized into three age groups, viz., <25 years, 25-40 years, and >40 years, and they were 41 (13.14%), 211 (67.62%), and 60 (19.23%) in numbers, respectively. According to profession or cadre, the HCP respondents in the study were divided into doctors, laboratory technicians, and nursing officers, and their numbers were 239 (76.60%), 12 (3.84%), and 61 (19.55%), respectively.

A routine practice of standard precautionary measures (face mask, hand hygiene, and social distancing) and wearing a personal protective equipment (PPE) kit during the care of COVID-19-positive or COVID-19-suspected patients was adopted by the majority of HCPs (n = 306; >98%). Three participants who did not adopt any preventive measures were two nursing officers and one doctor; out of them, two were in the age group of 25-40 years, and one was >40 years. Their values were statistically insignificant with respect to all the studied parameters. All three participants were male.

After the “routine preventive measures,” the most adopted measure by participant HCPs irrespective of profession, age, and gender was the consumption of allopathic drugs (n = 188; 60%), and the most non-drug preventive measures were the initiation of physical exercises and changes in dietary habits. Anti-COVID-19 measures in the category of drugs adopted by HCPs were prophylactic allopathic drugs (n = 188; 60%), homeopathic drugs (n = 37; 11.86%), and other Ayurveda, Yoga, Naturopathy, Unani, Siddha, and Homeopathy (AYUSH)/traditional medical system drugs (n = 37; 11.86%). Non-drug measures espoused by the HCPs were physical exercises (n = 145; 46.47%), increased sleep duration (n = 112; 35.89%), change in dietary habits (n = 133; 42.62%), and spiritual measures (n = 60; 19.23%).

During the observation of routine preventive practices in the hospital wards, it was observed that 100% of nursing officers and 99.2% of doctors adhered to these practices. However, only 66.7% of laboratory technicians followed the same practices, and the difference in adherence was statistically significant (p = 0.001). The same percentage (66.7%) of laboratory technicians consumed allopathic medicines to protect them from COVID-19 infection, which was almost similar among doctors (61.9%) and nursing officers (52.5%) (Table 1). The consumption of prophylactic allopathic drugs was the most adopted preventive measure after routine care in all the cadres of healthcare professionals.

**Table 1:** Adoption of preventive measure as per profession

Preventive measures	Profession						P-value
	Doctors (n = 239)		Laboratory technicians (n = 12)		Nursing officers (n = 61)		
	Frequency	%	Frequency	%	Frequency	%	
Routine preventive practice in ward	237	99.2	8	66.7	61	100.0	0.001
Allopathy	148	61.9	8	66.7	32	52.5	0.362
Homeopathy	25	10.5	2	16.7	10	16.4	0.384
Ayurveda	27	11.3	1	8.3	9	14.8	0.703
Physical exercises	109	45.6	7	58.3	29	47.5	0.676
Improvement in sleep duration	84	35.1	5	41.7	23	37.7	0.853
Change in diet habits	95	39.7	7	58.3	31	50.8	0.158
Spiritual measures	45	18.8	1	8.3	14	23.0	0.476
Did not adopt any measure	1	0.4	0	0.0	2	3.3	0.117

The least adopted measure by doctors and nursing officers was the consumption of Ayurvedic and homeopathic drugs alone. Similarly, the least preferred method by laboratory technicians was also Ayurvedic drugs and spiritual measures, making Ayurvedic and homeopathic drugs the least adopted preventive measures by HCPs (Table 2).

**Table 2:** Gender-wise adoption of preventive measure

Preventive measures	Gender				P-value
	Female (n = 114)		Male (n = 198)		
	Frequency	%	Frequency	%	
Routine preventive practice in ward	113	99.1	193	97.5	0.307
Allopathy	65	57.0	123	62.1	0.375
Homeopathy	17	14.9	20	10.1	0.207
Ayurveda	16	14.0	21	10.6	0.367
Physical exercises	39	34.2	106	53.5	0.001
Improvement in sleep duration	36	31.6	76	38.4	0.227
Change in diet habits	55	48.2	78	39.4	0.128
Spiritual measures	24	21.1	36	18.2	0.536
Did not adopt any measure	0	0.0	3	1.5	0.187

With respect to the genders of HCPs, both males and females adopted almost all the preventive measures as described for the professional categories. Overall, in the context of gender effect on preference for different COVID-19 preventive measures, there was no significant difference between males and females, which highlights high concern in both sexes to adopt some or other drug or non-drug measures among HCPs. The most preferred preventive measures adopted by female HCPs in sequential order after the routine measures in wards were allopathy drug consumption (57.0%), change in dietary habits (48.2%), physical exercises (34.2%), improvement in sleep duration (31.6%), and spiritual measures (21.1%), while male HCPs preferred using allopathy drugs (62.1%), physical exercises (53.5%), changes in diet habits (39.4%), improvement in sleep duration (38.4%), and spiritual measures (18.2%). Physical exercises were adopted by 106 (53.5%) males and 39 (34.2%) females, and the difference in the number of adoptions was statistically significant (p = 0.001). More female HCPs preferred changes in dietary habits (48.2%) over males (39.4%), but the statistical difference was not significant.

Participating HCPs were divided into three age groups, and the majority of the participants (n = 211; 67.62%) were in the age group of 25-40 years. When HCP data was scrutinized into the age bands, the number of participants who consumed allopathic medicines was 53.65% in the age group of <25 years, 60.66% in the age group of 25-40 years, and 63.33% in the age group of >40 years. Under the non-drug preventive measure category, the most commonly adopted measure was the improvement in sleep duration (31.7% in 25 years of age) and the initiation of physical exercises in 25 years of age.

The least adopted measures by HCPs in the age groups of 25 years and 25-40 years were homeopathic drug consumption (2.43% and 11%, respectively) and, in >40 years, Ayurvedic drug consumption (13.33%). Physical exercise adoption increased with age, with older HCPs (58.3%) adopting it more than younger HCPs (29.3%). Other adopted measures as per the age bands are described in Table 3.

**Table 3:** Adoption of preventive measures as per the age groups

Preventive measures	Age group						P-value
	<25 years (n = 41)		25-40 years (n = 211)		>40 years (n = 60)		
	Frequency	%	Frequency	%	Frequency	%	
Routine preventive practice in ward	41	100.0	205	97.2	60	100.0	0.231
Allopathy	22	53.7	128	60.7	38	63.3	0.474
Homeopathy	1	2.4	23	10.9	13	21.7	0.010
Ayurveda	4	9.8	25	11.8	8	13.3	0.765
Physical exercises	12	29.3	98	46.4	35	58.3	0.016
Improvement in sleep duration	13	31.7	76	36.0	23	38.3	0.791
Change in diet habits	12	29.3	95	45.0	26	43.3	0.174
Spiritual measures	6	14.6	41	19.4	13	21.7	0.673
Did not adopt any measure	0	0.0	2	0.9	1	1.7	0.701

The most common homeopathic medicine consumed by HCPs was arsenic album, and the most commonly consumed Ayurvedic medicines were ashwagandha, yashtimadhu, guduchi and pippali, and a polyherbal formulation (AYUSH-64). The number of participants who took the three types of drugs (allopathic, homeopathic, and Ayurvedic medicine) either together or at different points in time was nine (2.88%). The number of participants who did not adopt allopathic medicine but took a combination of homeopathic and Ayurvedic medicine was 11 (3.53%). A combination of allopathic and Ayurvedic medicine was taken by 19 participants (6.09%). The number of participants who consumed allopathic medicine along with homeopathic medicine was 16 (5.13%).

**Figure 1:**
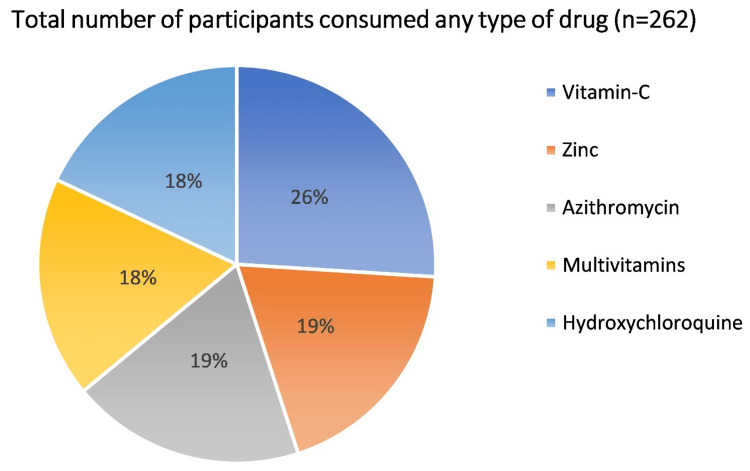
Pie chart representing the number of HCPs that consumed some or other drugs for the prevention of COVID-19 HCPs, healthcare providers; COVID-19, coronavirus disease 2019

Out of the total participants, 262 (83.97%) participants consumed some type of drug, and among them, 195 (74.42%) consumed vitamin C tablets. Thus, vitamin C was the most consumed drug among the participating HCPs. The frequency and order of oral drugs consumed by HCPs after vitamin C were zinc (142; 54.19%), azithromycin (139; 53.05%), multivitamins (135; 51.52%), and hydroxychloroquine (HCQ) (133; 50.76%) (Figure 1). Almost half of the participants who preferred HCQ took the drug as per the Indian Council of Medical Research (ICMR) guidelines, and their duration of consumption was eight weeks (27 participants) and four weeks (40 participants), and another half of the participants took the drug for an arbitrary duration. The commencement of physical exercises, improvement in dietary habits, and augmentation of sleep duration were adopted by 145 (46.47%), 133 (42.63%), and 112 (34.90%) participants, respectively. Most of the participants chose physical exercises in the form of brisk walking, cycling, dance, Zumba, aerobic exercise, and Yoga. On the other hand, 60 (19.23%) participants implemented spiritual measures. The number of participants who did not take any drugs were 105 (33.65%).

In HCPs who were taking drugs, 26 (9.92%) experienced few side effects, and the most common side effects were abdominal pain (12; 46.15%), nausea (11; 42.30%), constipation/diarrhea (10; 38.46%), and headache (nine; 34.61%). Tachycardia, palpitation, and vomiting were felt by three (11.53%) participants, and four (15.38%) participants felt dizziness, giddiness, and fainting. When the side effects of the consumed drugs were correlated with the total sample size (n = 262) who consumed any drug, abdominal pain, nausea, constipation or diarrhea, and headache were found in 4.58%, 4.19%, 3.81%, and 3.43%, respectively.

The total number of participants who also preferred non-drug measures either as the only option or in combination with drug measures was 161 (51.60%), and the most commonly opted non-drug measures were an increase in the use of indigenous spices (100; 32.05%), mild aerobic exercise (88; 28.20%), increase in sleep duration (64; 39.75%), and Yoga (46; 28.57%). The most commonly consumed indigenous spices were turmeric, garlic, tulsi, honey, and tea to boost immunity. Exercises preferred by the healthcare workers (HCWs) were Zumba dance, cycling, brisk walking, and jogging. Out of the participants who opted for non-drug measures, six (3.72%) noticed side effects, and the most common side effects were excessive tiredness (n = 3) and acidity or pain in the abdomen (n = 3).

Spiritual measures were adopted by 60 (around 20%) participants. More than half of the participants, 45 (72.58%), who adopted spiritual measures did meditation. Other spiritual measures opted for by the participants were the initiation of watching or reading religious materials (25; 41.67%), time devotion in worship (21; 35.0%), and hearing religious chants (satsang) (11; 18.33%). One of the participants fasted as a spiritual measure. The reasons for the adoption of any specific preventive measures by the HCPs are given in Table 4.

**Table 4:** Reasons for the adoption of preventive measures by the HCPs HCPs, healthcare providers; COVID-19, coronavirus disease 2019; ICMR, Indian Council of Medical Research

Reasons given by healthcare providers	Total number
Because of the fear of acquiring COVID-19 infection during the outbreak	188 (60.06%)
Because of the fear that my family may acquire COVID-19 infection from me	208 (66.64%)
Because ICMR has published an advisory to take pharmacological preventive measures for HCPs	115 (36.74%)

Out of the total participants, 102 (32.69%) got confused about choosing any of the preventive measures for different reasons (Table 5).

**Table 5:** Reasons for confusion in choosing any of the preventive measures

Reasons given by healthcare providers	Total number
Doubtful efficacy of various preventive measures (due to inadequate scientific evidence)	76 (24.28%)
Multiple sources of information regarding various preventive measures	71 (22.68%)
Fear of side effects with various drugs/non-drug measures or other modalities	34 (10.86%)
Multiple different advices advocated by friends/family members/colleagues	31 (9.90%)

Dietary modification was chosen by 133 (42.63%) participants, and the most common selection was an increase in intake of fruits and vegetables (n = 128; 96.24%). An increase in tea and warm water, foods containing vitamin C, proteins, spices, dry fruits, and immune-boosting supplements in the diet modification group was adopted by 120 (90.23%), 118 (88.72%), 109 (81.96%), 96 (72.18%), 63 (47.37%), and 61 (45.86%) patients, respectively.

Approximately half of the HCPs, i.e., 162 (51.92%), occasionally felt depression or apprehension due to the COVID-19 pandemic, and one-fourth of the participants, 80 (25.64%), never felt the same. Feelings of depression and apprehension were felt often and very frequently in 42 (13.41%) and 28 (8.94%) HCPs, respectively.

The most common consequence of apprehension in HCPs was irritation with small matters and a decrease in working efficiency, which were found in 107 (34%) and 94 (30%) participants, respectively. The initiation of shouting at family members, friends, and colleagues was found in 42 (13.41%) participants.

Before the initiation of this study, 208 (66.45%) participants had undergone reverse transcription-polymerase chain reaction (RT-PCR) for COVID-19 testing at different points in time. Although 32 (10.22%) HCPs were found laboratory-confirmed COVID-19-positive, 126 (40.25%) participants had experienced symptoms such as fever, headache, sore throat, dry cough or cold, shortness of breath, loss of taste and smell sensation, tiredness, diarrhea, and chest pain before the initiation of this study.

## Discussion

Coronavirus disease 2019 (COVID-19) was one of the most dreaded infectious diseases that affected the lifestyle, living conditions, and mental status of HCPs across the world. Out of the total participants (n = 306), seven did not use routine standard precautionary measures (face mask, hand hygiene, and social distancing) and a PPE kit. The reasons might have been a lack of awareness or a boost to practice PPE, the nonavailability of proper PPE kits at some working places, or a casual attitude due to duty in non-COVID-19 areas of the hospital. A few might have used it intermittently and not as a routine. The CDC, WHO, and other governing bodies have released a list of preventive measures to be taken by HCPs to protect themselves from COVID-19 infections [3,4]. But in the first wave of the pandemic, due to a lack of evidence-based literature, HCPs were forced to adopt preventive measures mostly based on ancient and acquired wisdom. The COVID-19 pandemic made the world realize that HCWs must keep themselves safe while taking care of their patients, and the WHO expressed the same in a health worker safety charter on September 17, 2020.

According to a cross-sectional descriptive study (2020), in which medical students from six medical schools in Jordan were assessed for their knowledge, attitude, and practice of precautionary measures, it was found that >80% of them adopted social isolation, frequent hand washing, and enhanced personal hygiene measures as their first line of defense against COVID-19 infection [5]. In a similar study done on dental students, 93.0% accepted the importance of hand hygiene, face masks, and social distancing, and out of them, 63.0% of students were familiar with the term PPE [6]. In another study done with 1134 HCPs, including doctors, nursing officers, midwives, and interns, from six medical hospitals in Ethiopia, 93.0% were adopting hand washing and face masks as preventive measures, and the mean (median) worry score about the COVID-19 crisis was 2.37 (3.0), on a 1-3 scale, with 1 being not worried and 3 highly worried. Out of them, 88.0% of HCPs expressed the risk of acquiring COVID-19 infection, and 91.0% also expressed worry about the potential risk of infection to their family [7].

Adopting good clinical preventive measures by HCPs is imperative for their protection. In a study done with 238 HCPs at Dilla University Hospital, 56.3% (95% CI: 50-60.3) were adopting good COVID-19 prevention practices [8]. But in the current study, the quality of preventive measures was not assessed. A similar study expressed the gap in HCPs’ adoption of preventive measures [9]. In some of the studies, it is evident that HCPs have an equal or greater tendency to acquire COVID-19 infection as compared to the public infectivity data [10]. According to a systematic review, the identification of the best preventive measure was difficult and required more studies for a definitive conclusion. There are mixed approaches adopted by HCPs to protect themselves [11].

Although the homeopathic medications consumed by HCPs during the onset of the first COVID-19 wave as preventive measures were out of fear and a lack of literature, now, case series and many other studies from different geographical areas have been published to showcase the effectiveness of their therapeutic potential [12-15]. Few studies showed its utility in decreasing the duration of illness when used as an adjunctive [14]. Although the Government of India’s AYUSH Ministry advised people to use homeopathy medicine to prevent COVID-19 infections during the first arrival of COVID-19 in India, several countries such as Australia, France, and Britain declared it pseudoscience.

A study on 22 adults who were regularly doing exercises at the gymnasium revealed that continuing exercises at home during lockdown helped them conquer fitness and psychological issues [16]. In our current study, 145 (46.32%) participants preferred doing some form of physical exercise to boost their immunity.

A cross-sectional study done between May 4 and July 31, 2020, revealed the extent and pattern of the use of AYUSH-based measures by the Indian population. Using a mobile-based application, it was reported that during the COVID-19 pandemic, 616,295 (85.2%) participants had been using AYUSH measures to safeguard their health [17]. In the current study too, immunity-promoting interventions from the ancient traditional systems of medicine were adopted by 23.64% of the participants. In a Chinese study, frontline epidemic prevention workers were consuming high-salt, fried food and had the habit of smoking before the COVID-19 pandemic, and most of them expressed their willingness to quit [18]. In the present study, 161 (51.43%) participants adopted dietary modifications used by the HCPs as a preventive measure against COVID-19. In the reviewed literature, most emphasize the consumption of vegetables, fruits, and whole grains, and 31% of them describe the role of zinc and vitamins C, A, and D to maintain the immune system, but strong evidence is still lacking [19]. Like other literature, in the current study too, the most common (69.64%) dietary modification was the increased consumption of fruits and vegetables.

To overcome distress and anxiety during the COVID-19 pandemic, 73 (23.32%) HCPs in the current study adopted spiritual measures.

Many HCPs faced psychological distress during the COVID-19 pandemic as they were more susceptible to emotional, behavioral, and psychological stress due to their duty to maintain a balance between patient care and taking care of their loved ones [20,21]. In a systematic review of literature searched between December 2019 and April 12, 2020, it was found that the lowest reported prevalence of anxiety, depression, and stress among HCPs was 24.1%, 12.1%, and 29.8%, respectively, and the highest prevalence was 67.55%, 55.89%, and 62.99%, respectively [22]. In our study, as revealed by participant HCWs, depression and apprehension were felt “often” and “very frequently” in 42 (13.41%) and 28 (8.94%) HCPs, respectively, but no severity scoring system to assess the psychological impact could be used in it.

A significant limitation of this research study is the reliance on self-reported surveys, coupled with the absence of follow-up assessments to ensure participant anonymity. Consequently, the accurate evaluation of the effectiveness of each preventive measure utilized and reported in the study was hindered.

## Conclusions

Fear related to the COVID-19 pandemic imposed on the HCPs the obligation to use all the possible and available preventive measures despite the lack of evidence of their actual benefits. Routine preventive practices of the hospital were followed by only 66.7% of the laboratory technicians, which was significantly lower than the doctor (99.2%) and nursing officer (100%) groups. After the routine preventive or standard measures in the wards or other designated areas, the most adopted measure by all HCPs, irrespective of profession, age, or gender, was the consumption of allopathic drugs prevalent at that time. In non-drug measures, physical exercises were adopted significantly more by males (53.5%) than females (34.2%). The preference of female HCPs was for changes in dietary habits. Among HCPs practicing allopathy, about 40% adopted other AYUSH measures, such as homeopathic and Ayurvedic drugs. Physical exercise adoption rate increased with age, as it was more adopted by HCPs with ages of >40 years over younger HCPs with ages of <25 years (58.6% versus 29.3%).
